# Simulating neuronal development: exploring potential mechanisms for central nervous system metastasis in acute lymphoblastic leukemia

**DOI:** 10.3389/fonc.2023.1331802

**Published:** 2024-01-04

**Authors:** Ziping Li, Zhi Guo, Haitao Xiao, Xuexing Chen, Wei Liu, Hao Zhou

**Affiliations:** ^1^ Institute of Hematology, Union Hospital, Tongji Medical College, Huazhong University of Science and Technology, Wuhan, China; ^2^ Department of Hematology, Huazhong University of Science and Technology Union Shenzhen Hospital, Shenzhen, China; ^3^ Department of Anatomy, Tongji Medical College, Huazhong University of Science and Technology, Wuhan, China; ^4^ Key Laboratory of Neurological Diseases of Ministry of Education, Tongji Medical College, Huazhong University of Science and Technology, Wuhan, China; ^5^ Institute of Hematology, Xiangyang Central Hospital, Affiliated Hospital of Hubei University of Arts and Science, Xiangyang, China

**Keywords:** acute lymphoblastic leukemia (ALL), metastasis, central nervous system (CNS), neuronal development, differentially expressed genes

## Abstract

**Background:**

Acute lymphoblastic leukemia (ALL) is prone to metastasize to the central nervous system (CNS), which is an important cause of poor treatment outcomes and unfavorable prognosis. However, the pathogenesis of CNS metastasis of ALL cells has not been fully illuminated. Recent reports have shed some light on the correlation between neural mechanisms and ALL CNS metastasis. These progressions prompt us to study the relationship between ALL central nervous system metastasis and neuronal development, exploring potential biomarkers and therapeutic targets of CNS metastasis.

**Materials and methods:**

ALL central nervous system metastasis- and neuronal development-related differentially expressed genes (DEGs) were identified by analyzing gene expression datasets GSE60926 and GSE13715. Target prediction and network analysis methods were applied to assess protein–protein interaction networks. Gene Ontology (GO) terms and pathway enrichment for DEGs were assessed. Co-expressed differentially expressed genes (co-DEGs) coupled with corresponding predicted microRNAs (miRNAs) were studied as well. Reverse transcription–polymerase chain reaction (RT–PCR) and flow cytometry were employed for the validation of key co-DEGs in primary ALL cells. Furthermore, ALL cells were treated with a vascular endothelial growth factor (VEGF) inhibitor to block neuronal development and assess changes in the co-DEGs.

**Results:**

We identified 216, 208, and 204 DEGs in ALL CNS metastasis specimens and neuronal development samples (GSE60926 and GSE13715). CD2, CD3G, CD3D, and LCK may be implicated in ALL CNS metastasis. LAMB1, MATN3, IGFBP3, LGALS1, and NEUROD1 may be associated with neuronal development. Specifically, four co-DEGs (LGALS1, TMEM71, SHISA2, and S100A11) may link ALL central nervous system metastasis and neuronal development process. The miRNAs for each co-DEG could be potential biomarkers or therapeutic targets for ALL central nervous system metastasis, especially hsa-miR-22-3p, hsa-miR-548t-5p, and hsa-miR-6134. Additionally, four co-DEGs (LGALS1, TMEM71, SHISA2, and S100A11) were validated in CNS-infiltrated ALL cells. The VEGF inhibitor demonstrated a suppressive effect on mRNA and protein expression of key co-DEGs.

**Conclusion:**

The bioinformatic survey and key gene validation suggest a possible correlation between ALL CNS metastasis and the neuronal development process. Simulating the neuronal development process might be a possible strategy for CNS metastasis in ALL. LGALS1, TMEM71, SHISA2, and S100A11 genes are promising and novel biomarkers and targets in ALL CNS metastasis.

## Background

Acute lymphoblastic leukemia (ALL) is a malignancy that impacts the lymphoid lineage of hematopoietic cells and is distinguished by the excessive proliferation of immature lymphocytes (1). Specifically, ALL exhibits a notable tendency toward metastasizing to the central nervous system (CNS) (2). In spite of the advancements achieved in the management of ALL, individuals with unfavorable prognoses exhibit a higher incidence of CNS involvement. Moreover, the occurrence of late CNS relapse is often associated with a fatal outcome, thereby presenting a substantial obstacle in the attempt for a cure for these patients. The prognosis for adult patients who encounter CNS relapse is highly unfavorable, with a median overall survival (OS) of 6 months and a projected 5-year OS of zero (3). Additionally, even though routine diagnostic methods often fail to detect the presence of CNS involvement, early autopsy studies have already demonstrated that half of the patients suffer from submicroscopic CNS disease (4), underscoring the necessity for a suitable approach to investigate the entry of leukemia cells into the CNS.

At the time of diagnosis, the central nervous system is frequently affected as an extramedullary site in all cases. The CNS acts as a sanctuary site, contributing to the occurrence of relapse (5). The restricted permeability of the blood–brain barrier (BBB) and the existence of a blood–tumor barrier (BTB) limit the accessibility of conventional medications to the central nervous system, leading to resistance to treatment and a poor prognosis. Significant progress has been made in enhancing the survival rate of ALL patients through the implementation of intrathecal chemotherapy, high-dose systemic chemotherapy, and/or cranial radiotherapy, specifically targeting the central nervous system. Despite their lack of specificity, CNS-targeted therapies can lead to adverse effects such as seizures, leukoencephalopathy, and delayed neurodevelopmental effects (6).

The limitations of current diagnostic methods and CNS-directed therapy prompt us to uncover mechanisms by which ALL cells infiltrate the CNS. Recent reports have shed some light on the correlation between neural mechanisms and ALL CNS metastasis (2). In this study, we identified co-expressed differentially expressed genes (co-DEGs) of ALL central nervous system metastasis and neuronal development and elucidated molecular mechanisms and pathology of ALL central nervous system metastasis-related DEGs and neuronal development-related DEGs. Furthermore, we provided a bioinformatic analysis of DEGs and predicted microRNAs (miRNAs), which regulate the co-expression of DEGs. Finally, we validated four key DEGs in primary ALL cells and provided a potential and novel strategy against CNS metastasis.

## Materials and methods

### Patient samples and ethics statement

After written informed consent was obtained, leukemic cells were gathered from ALL patients with CNS metastasis. By separating mononuclear cells on a Ficoll density gradient (Sigma-Aldrich, St. Louis, MO, USA), cell suspensions containing >80% leukemic cells were produced. The present study was approved by the Ethics Committee of Tongji Medical College, Huazhong University of Science and Technology, and performed in strict compliance with the Declaration of Helsinki.

### Gene Expression Omnibus datasets

We screened Gene Expression Omnibus (GEO) datasets GSE60926 and GSE13715 according to the following inclusion criteria: 1) *Homo sapiens* ALL central nervous system metastasis specimens, 2) expression profiling by array, 3) performed on the GPL570 platform ([HGU133_Plus_2] Affymetrix Human Genome U133 Plus 2.0 Array), and 4) ≥8 samples and downloaded from GEO (7). The GSE60926 dataset includes 20 specimens from bone marrow (BM) and eight specimens from cerebrospinal fluid (CSF) of ALL patients. The GSE13715 dataset contains four and three specimens from neural progenitor cells (NPCs) before differentiation and four and three specimens after differentiation cultured on 2D substrates and 3D porous polystyrene scaffolds, respectively. We used the identified two datasets to analyze the relationship between ALL central nervous system metastasis and neuronal development.

### Data processing

We identified DEGs between BM samples and CSF in ALL, with the DEGs about neuronal differentiation, from R using the packages “affy”, “affyPLM”, and “limma” (8). Gene expression values of the |log2 FC| > 2 and p-value <0.05 were significant. Additionally, we calculated and made Venn diagrams for co-DEGs for ALL central nervous system metastasis- and neuronal development-related DEGs.

### Protein–protein interaction network construction

Protein–protein interaction (PPI) networks of ALL central nervous system metastasis- and neuronal development-related DEGs were analyzed using the STRING software (9) and obtained the analytic results with a confidence score >0.7. Subsequently, the results were performed using the Cytoscape software (10) to visualize molecular interaction network node degrees.

### Functional enrichment analysis

Gene Ontology (GO) and Kyoto Encyclopedia of Genes and Genomes (KEGG) pathway enrichment analyses of ALL central nervous system metastasis- and neuronal development-related DEGs were carried out using the Database for Annotation, Visualization and Integrated Discovery (DAVID) (11) and Reactome databases (12). GO terms and KEGG pathways of biological functions were considered to be significantly enriched if p < 0.05. At the same time, the different bio functions of ALL central nervous system metastasis- and neuronal development-related DEGs in biological process (BP), molecular function (MF), and cellular component (CC) were presented using the DAVID database. Moreover, the AmiGO2 database (13) was used to analyze the GO terms for the selected co-DEGs to verify the accuracy and annotate bio-functions of identified co-DEGs.

### Predict the co-DEG-relevant miRNAs

We applied the online prediction tools microRNA Data Integration Portal (mirDIP) (14), microRNA Target Prediction Database (miRDB) (15), TargetScanHuman (16), and DIANA TOOLS (17) to predict potential microRNAs that could target the co-DEGs. We obtained the candidate microRNAs according to higher predicted scores for ≥3 prediction tools for each co-DEG.

### Identification of co−DEGs associated with other nervous system diseases

The Comparative Toxicogenomics Database (CTD) (http://ctdbase.org/) (18) provides manually curated information about chemical–gene/protein interactions, chemical–disease, and gene–disease relationships, which was used to find gene–disease interactions to generate expanded networks and predict novel associations. These data were used to analyze relationships between the co-DEGs and hematological or nervous system diseases.

### Cell culture and treatment

Primary ALL cells separated using density gradient centrifugation with Ficoll-Hypaque were cultured in 24-well plates at 2 × 10^6^ cells/ml at 37°C with 5% CO_2_ in the following: RPMI 10 (control medium) or vascular endothelial growth factor (VEGF) inhibitor thalidomide (40 μM, Sigma-Aldrich).

### Flow cytometric measurement

All samples were detected using the flow cytometry analyzer BD FACSCanto II (Becton Dickinson, Franklin Lakes, NJ, USA). Measured data were analyzed using the BD CellQuest software (Becton Dickinson, USA). Anti-LGALS1 (ab228184) and anti-S100A11 (ab236123) for surface staining of leukemia cells were obtained from Abcam (Cambridge, UK). The procedure was as previously described (19).

### Reverse transcription–polymerase chain reaction

For reverse transcription–polymerase chain reaction (RT–PCR) experiments, RNA was isolated using TRIzol reagent (Invitrogen, Carlsbad, CA, USA). cDNA was produced from 1 μg RNA using the First Strand cDNA synthesis kit (TaKaRa, Maebashi, Japan). PCR was then performed on 0.5 μl cDNA. The procedures were conducted according to the instructions by the manufacturers. The PCR primers (5′→3′) used were LGALS1 forward (Fw): CTGTGCCTGCACTTCAACC; TMEM71 Fw: GATGTCAACACCAGTAGCAAGT; SHISA2 Fw: GGAGACCATCCCCATGATCC; S100A11 Fw: CTGAGCGGTGCATCGAGTC; LGALS1 reverse (Rv): CATCTGGCAGCTTGACGGT; TMEM71 Rv: TGGTAACCATCCAGGGAATCA; SHISA2 Rv: AGCACAGAGAAATTCGTGGGC; S100A11 Rv: TGTGAAGGCAGCTAGTTCTGTA.

### Statistical analysis

Statistical analyses were conducted using SPSS 20.0 (IBM Corp., Armonk, NY, USA) and GraphPad Prism 8.0.2 (GraphPad Software, San Diego, CA, USA). Student’s t-test and analysis of variance were applied to compare continuous variables between the two groups or among multiple groups. Categorical variables were analyzed using the chi-square test, continuity correction test, or Fisher’s exact test. Statistical significance was set at p-values <0.05.

## Results

### Identification of DEGs

**Figure 1:**
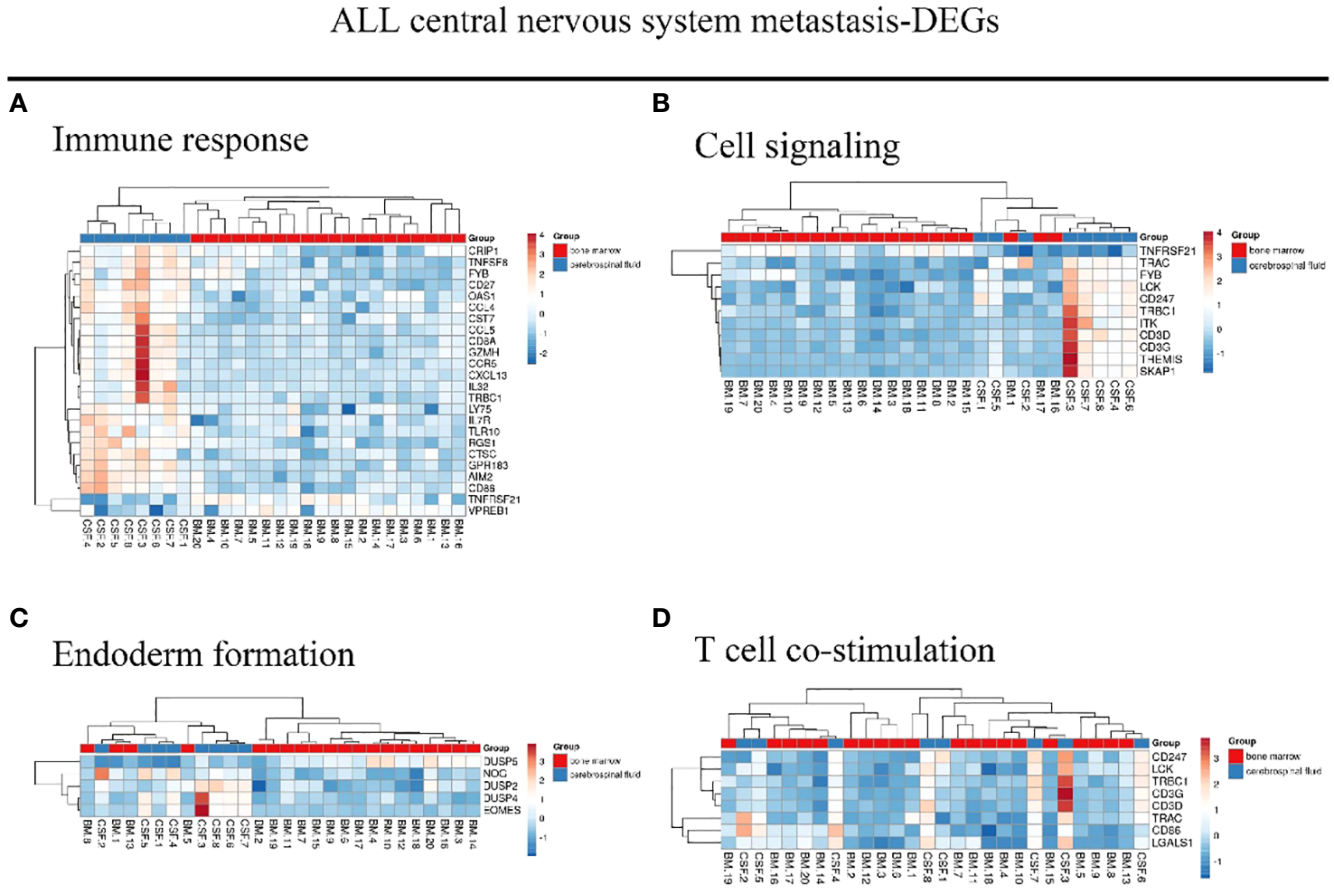
Hierarchical clustering analysis of ALL central nervous system metastasis-related differentially expressed genes. **(A–D)** Results of hierarchical clustering analysis for DEG expression in relation to immune response, cell signaling, endoderm formation, and T-cell co-stimulation. Red, greater expression. Blue, less expression. BM, bone marrow; CSF, cerebrospinal fluid; ALL, acute lymphoblastic leukemia; DEG, differentially expressed gene.

**Figure 2:**
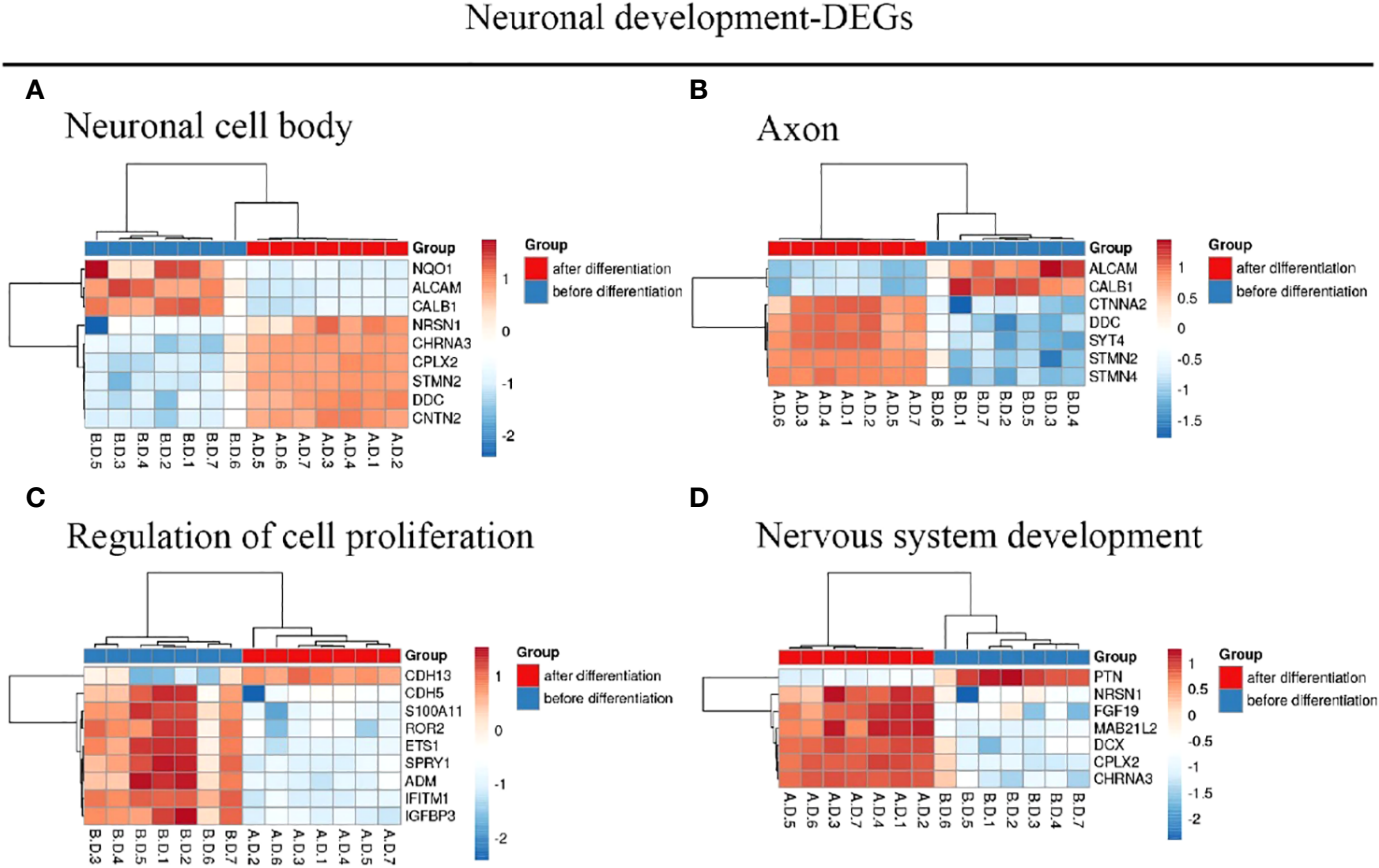
Hierarchical clustering analysis of neuronal development-related DEGs. **(A–D)** Results of hierarchical clustering analysis for DEG expression in relation to neuronal cell body, axon, regulation of cell proliferation, and nervous system development. Red, greater expression. Blue, less expression. AD, after differentiation; BD, before differentiation; DEGs, differentially expressed genes.

We identified 216 DEGs in the GSE60926 dataset, and we identified a total of 208 and 204 DEGs in the 2D substrate specimens and 3D porous polystyrene scaffold specimens in the GSE13157 dataset, respectively. Furthermore, we defined 82 co-expressed DEGs in the 2D specimens and 3D specimens mentioned above as the neuronal development DEGs. We conducted a cluster analysis of ALL central nervous system metastasis-related DEGs in relation to immune response and cell signaling, endoderm formation, and T-cell co-stimulation for gene expression, and these data appear in **Figure 1** and **Supplementary Table S1**. Both **Figure 2** and **Supplementary Table S2** show the gene expression value in relation to the neuronal cell body, axon, regulation of cell proliferation, and nervous system development above the neuronal development-related DEGs.

### Functional enrichment in co−DEGs

**Figure 3:**
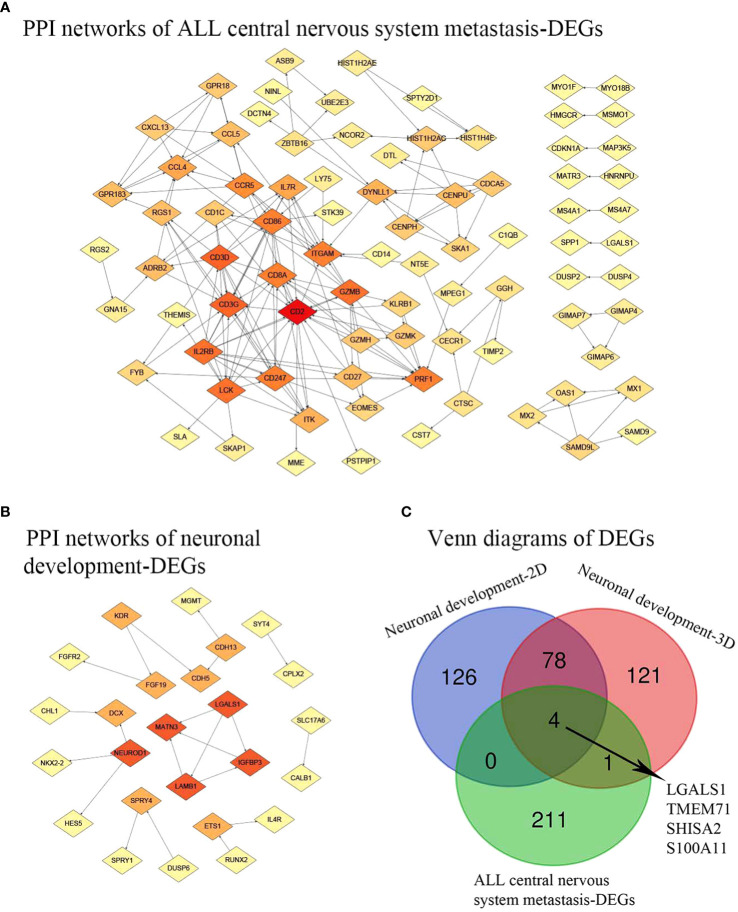
PPI network and Venn diagrams. **(A)** PPI network of ALL central nervous system metastasis-related DEGs. **(B)** PPI network of neuronal development-related DEGs. Red, greater degree. Yellow, lesser degree. **(C)** Venn diagrams of DEGs. Neuronal development-2D, differential genes found in specimens cultured on 2D substrates; Neuronal development-3D, differential genes found in specimens on 3D porous polystyrene scaffolds; PPI, protein–protein interaction; ALL, acute lymphoblastic leukemia; DEGs, differentially expressed genes.

**Figure 3C** depicts the differentially expressed genes associated with both central nervous system metastasis and neuronal development, as well as the genes that are co-expressed with them. Interestingly, four co-expressed DEGs, including galectin 1 (LGALS1), transmembrane protein 71 (TMEM71), shisa family member 2 (SHISA2), and S100 calcium-binding protein A11 (S100A11), were observed. The AmiGO database was used to confirm GO term enrichment related to biological processes, molecular functions, and cellular components, and co-DEGs were associated with various processes as indicated in **Table 1**.

**Table 1:** The Gene Ontology (GO) term enrichment for the co-expressed genes of the acute lymphoblastic leukemia (ALL) central nervous system metastasis and neuronal development.

Gene/product	GO class (direct)	Evidence	Evidence with	Reference
LGALS1	T-cell co-stimulation	IEA	UniProtKB:P16045	GO_REF:0000107
	Protein binding	IPI	UniProtKB:P08575	PMID:10369126
	Response to drug	IEA	UniProtKB:P11762	GO_REF:0000107
		IEA	UniProtKB:P11762	GO_REF:0000107
	Extracellular matrix	ISS	UniProtKB:Q49I35	PMID:22261194
		HDA		PMID:23979707
TMEM71	Integral component of membrane	IEA	UniProtKB-KW : KW-0812	GO_REF:0000043
	Mitochondrion	IDA		GO_REF:0000052
SHISA2	Negative regulation of fibroblast growth factor receptor signaling pathway	IBA	PANTHER : PTN001264939	PMID:21873635
S100A11	Calcium ion binding	IBA	PANTHER : PTN007521293	PMID:21873635
	Signal transduction	TAS		PMID:16130169
	Negative regulation of cell proliferation	TAS		PMID:10851017
	Protein homodimerization activity	IDA		PMID:10913138
	Protein binding	IPI	UniProtKB:P04271	PMID:10913138
		IBA	PANTHER : PTN000181483	PMID:21873635
	Extracellular space	IBA	PANTHER : PTN002615769	PMID:21873635
		HDA		PMID:23580065
	Calcium-dependent protein binding	IBA	PANTHER : PTN007521293	PMID:21873635
		IDA		PMID:10913138

### PPI network analysis and functional GO term and pathway enrichment analyses

A total of 195 nodes were identified from the PPI network of DEGs associated with central nervous system metastasis in acute lymphoblastic leukemia. Additionally, 82 nodes were identified from the PPI network of DEGs associated with neuronal development. These findings are presented in **Figure 3**. In the context of central nervous system metastasis in ALL, the following five hub nodes were identified as significant: CD2 (degree = 19), CD3G (degree = 13), CD3D (degree = 13), lymphocyte-specific protein tyrosine kinase (LCK; degree = 12), and interleukin 2 receptor subunit beta (IL2RB; degree = 12). These genes are considered to play a crucial role as hub genes in the aforementioned condition. Additionally, the hub genes, involved in laminin subunit beta 1 (LAMB1; degree = 3), matrilin 3 (MATN3; degree = 3), insulin-like growth factor binding protein 3 (IGFBP3; degree = 3), galectin 1 (LGALS1; degree = 3), and neuronal differentiation 1 (NEUROD1; degree = 3), were demonstrated in neuronal development-related DEGs with a higher degree.

**Figure 4:**
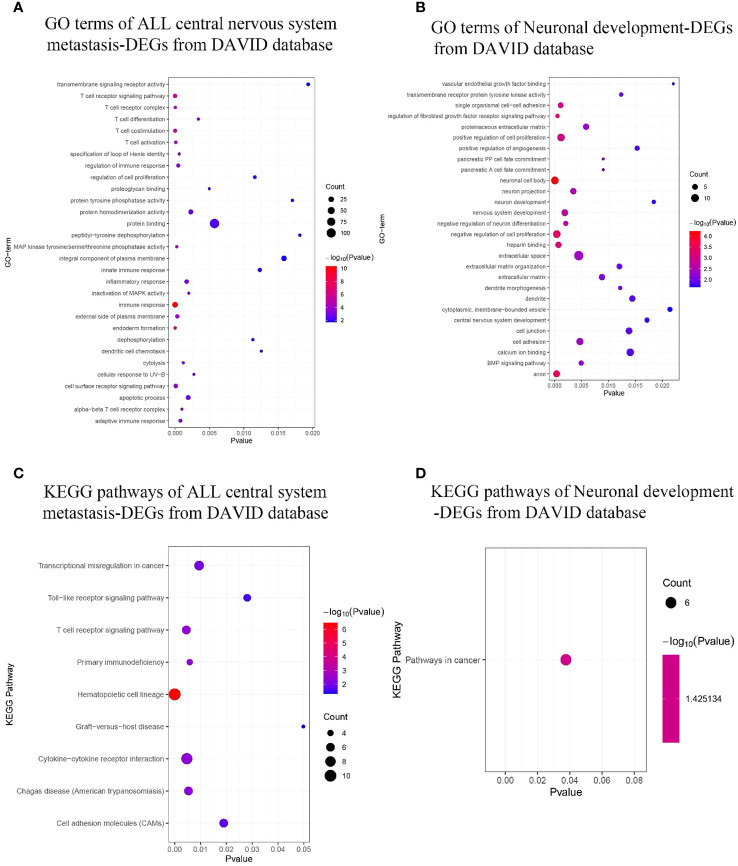
GO terms and KEGG pathway enrichment. **(A, B)** ALL central nervous system metastasis- and neuronal development-related GO term enrichment for DEGs. **(C, D)** KEGG pathway of ALL central nervous system metastasis- and neuronal development-related DEGs. Dot sizes represent counts of enriched DEGs, and dot colors represent negative Log10-p values. GO, Gene Ontology; KEGG, Kyoto Encyclopedia of Genes and Genomes; ALL, acute lymphoblastic leukemia; DEGs, differentially expressed genes.

Using the DAVID database, the top 3 GO terms related biological processes among ALL central nervous system metastasis-related DEGs were primarily associated with immune response (Fold Enrichment: 5.60; p-value: 4.60E−11), T-cell receptor signaling pathway (Fold Enrichment: 7.30; p-value: 2.66E−06), and endoderm formation (Fold Enrichment: 40.92; p-value: 4.71E−06). There was a significant correlation in T-cell receptor complex (Fold Enrichment: 27.36; p-value: 2.76E−05), external side of the plasma membrane (Fold Enrichment: 4.62; p-value: 3.22E−04), and alpha–beta T-cell receptor complex (Fold Enrichment: 59.10; p-value: 9.94E−04) in relation to cellular components. In addition, the terms related to molecular functions were mainly involved in mitogen-activated protein (MAP) kinase tyrosine/serine/threonine phosphatase activity (Fold Enrichment: 31.10; p-value: 2.48E−04), protein homodimerization activity (Fold Enrichment: 2.35; p-value: 0.002), and proteoglycan binding (Fold Enrichment: 27.57; p-value: 0.004). With respect to neuronal development-related DEGs, the biological processes in terms of negative regulation of cell proliferation (Fold Enrichment: 4.96; p-value: 4.15E−04), regulation of fibroblast growth factor receptor signaling pathway (Fold Enrichment: 81.78; p-value: 5.56E−04), and single organismal cell–cell adhesion (Fold Enrichment: 10.80; p-value: 0.001) were significantly enriched. Additionally, the top 3 GO terms related to cellular components were associated with neuronal cell body (Fold Enrichment: 6.59; p-value: 5.98E−05), axon (Fold Enrichment: 7.27; p-value: 3.78E−04), and neuron projection (Fold Enrichment: 5.84; p-value: 0.003). Similarly, the terms of heparin binding-related (Fold Enrichment: 8.33; p-value: 7.27E−04), transmembrane receptor protein tyrosine kinase activity-related (Fold Enrichment: 17.54; p-value: 0.012), and calcium ion binding-related (Fold Enrichment: 2.79; p-value: 0.014) molecular functions were primarily enriched (as shown in **Figures 4A, B**; **Supplementary Table S3**).

**Figure 5:**
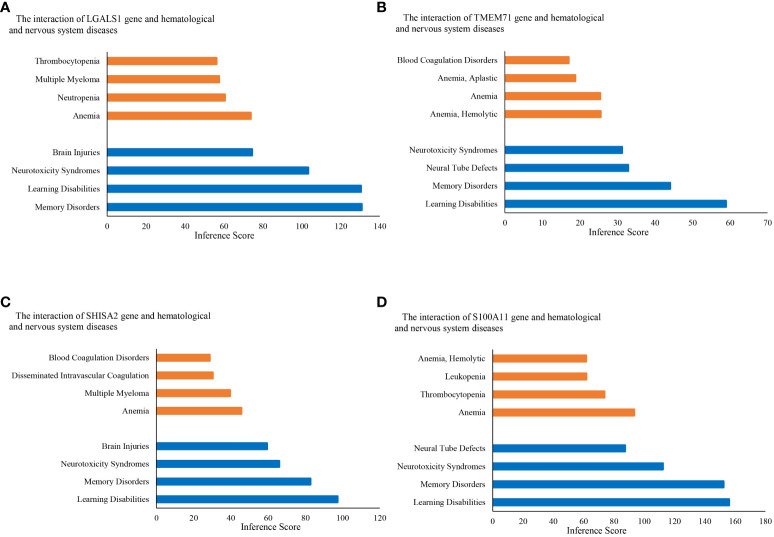
Relationship to hematological diseases and nervous system diseases related to co-expressed genes based on the CTD. Relationship between diseases and co-expressed genes **(A–D)**. CTD, The Comparative Toxicogenomics Database.

KEGG pathway analysis data from the DAVID database are shown in **Figures 4C, D**. The results suggest that the ALL central nervous system metastasis-related DEGs were mainly enriched in pathways of hematopoietic cell lineage (p-value: 3.46E−07), T-cell receptor signaling pathway (p-value: 0.005), and cytokine–cytokine receptor interaction (p-value: 0.005). However, there is only one KEGG term pathway in cancer (p-value: 0.04) that was significantly enriched in neuronal development-related DEGs (as shown in **Figure 4**; **Supplementary Table S4**). Additionally, KEGG pathway enrichments from the Reactome database also appeared in **Supplementary Table S4**. The CTD showed that co-DEGs targeted several hematological diseases or nervous system diseases, and these data appear in **Figure 5** and **Supplementary Table S5**.

### Identification of functional and pathway enrichment among predicted miRNAs and co−DEGs

The application of bioinformatic tools including mirDIP, miRDB, TargetScan, and DIANA enabled the identification of the top 5 miRNAs that target each co-DEG associated with central nervous system metastasis and neuronal development in ALL. The results of this prediction analysis are presented in **Table 2**. These data provide insights into the association between predicted miRNAs and central nervous system metastasis in relation to neuronal development in patients with ALL.

**Table 2:** The Gene Ontology (GO) terms among predicted microRNAs (miRNAs) and co-expressed differentially expressed genes (co-DEGs).

Genes	Predicted miRNAs	Category		p-Value
LGALS1	hsa-miR-22-3p	GO terms	Positive regulation of erythrocyte aggregation	0.002
			Lactose binding	0.002
			Plasma cell differentiation	0.002
			Galactoside binding	0.005
			Multicellular organismal response to stress	0.005
TMEM71	hsa-miR-656-3p		NA	NA
	hsa-miR-103a-3p			
	hsa-miR-186-5p			
	hsa-miR-107			
	hsa-miR-767-5p			
SHISA2	hsa-miR-3163		NA	NA
	hsa-miR-361-5p			
	hsa-miR-3662			
	hsa-miR-494-3p			
	hsa-miR-550a-5p			
S100A11	hsa-miR-548t-5p	GO terms	S100 protein binding	0.012
	hsa-miR-6134		Negative regulation of DNA replication	0.012
			Calcium-dependent protein binding	0.021
			Ruffle	0.022

### Testing co-DEGs in CNS-metastasized ALL cells and their changes under VEGF inhibitor treatment

**Figure 6:**
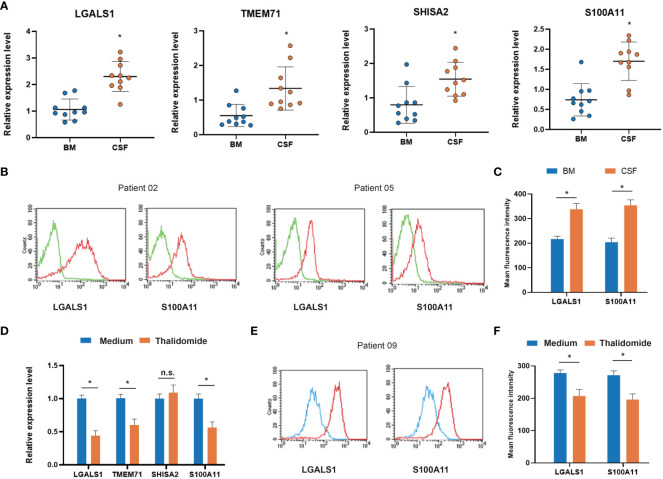
Expression of co-DEGs in primary ALL cells from CNS metastasis patients. **(A)** RT–PCR analysis of co-DEGs comparing bone marrow-derived and cerebrospinal fluid-derived ALL cells (n = 10). **(B)** Representative flow cytometry images of membrane-bound co-DEGs in bone marrow-derived and cerebrospinal fluid-derived ALL cells (n = 10; green, negative control). **(C)** Box-plot analysis of relative fluorescence intensity (RFI) of membrane-bound co-DEGs. **(D)** RT–PCR analysis of co-DEG changes in primary ALL cells treated with VEGF inhibitor thalidomide (n = 5). **(E)** Representative flow cytometry images of membrane-bound co-DEGs in primary ALL cells treated with VEGF inhibitor (n = 5; blue, thalidomide treated; red, medium control). **(F)** Box-plot analysis of RFI of co-DEGs after thalidomide treatment (* *p* < 0.05). BM, bone marrow; CSF, cerebrospinal fluid; VEGF, vascular endothelial growth factor; co-DEGs, co-expressed differentially expressed genes; ALL, acute lymphoblastic leukemia; CNS, central nervous system; RT–PCR, reverse transcription–polymerase chain reaction.

To validate the *in silico* finding, we applied RT–PCR to test the four co-DEGs (LGALS1, TMEM71, SHISA2, and S100A11) in CNS-metastasized ALL cells. The demographic and clinical characteristics of the ALL patients are presented in **Supplementary Table 6**. All four co-DEGs were significantly upregulated in CSF-derived ALL cells, compared with bone marrow-derived ALL cells (**Figure 6A**). To clarify the changes in protein level, we focused on two membrane-bound proteins (LGALS1 and S100A11) and assessed them using flow cytometry. LGALS1 and S100A11 proteins were increased in CSF-derived ALL cells, compared with bone marrow-derived ALL cells (**Figures 6B, C**). Furthermore, we used a VEGF inhibitor (thalidomide, 40 μM), which can hamper the neuronal development process (19), to treat primary cultured ALL cells. We found that LGALS1 and S100A11 were decreased after thalidomide treatment at both mRNA and protein levels (**Figures 6D–F**). The analysis of GEO data (GSE5820) showed that LGALS1 increased in poor clinical outcome patients (**Supplementary Figure 1**). These data verified four co-DEGs in CNS-metastasized ALL cells and implied a potential strategy for controlling CNS metastasis.

## Discussion

ALL has a marked propensity to metastasize to the central nervous system (19). The involvement of the central nervous system has long been recognized as a significant barrier to achieving successful long-term treatment outcomes and has been identified as a prominent factor contributing to mortality rates in the context of a more regulated systemic illness (20). Nevertheless, the mechanism by which leukemia cells invade the central nervous system remains uncertain. Various mechanisms have been proposed based on several lines of clinical or experimental evidence. Recent research has revealed that leukemia cells have the ability to migrate into the CNS by means of vessels, which are recognized for their role in guiding the pathfinding of neuronal progenitor cells within the CNS (19). These reports proposed a possible and functional relationship between neural mechanisms and ALL CNS metastasis. Based on these findings, we hypothesized that there might be a relationship between ALL CNS metastasis and neuronal development.

In the present study, we collected the gene expression profiles of ALL CNS metastasis GSE60926 and neuronal development GSE13715 from GEO. We identified a total of 216 genes differentially expressed between the primary site of bone marrow and the cerebrospinal fluid, as well as 82 differentially expressed genes before and after the differentiation of neurocytes. We identified four co-expression genes—LGALS1, TMEM71, SHISA2, and S100A11—between ALL CNS metastasis and neuronal development. Additionally, we constructed a PPI network and found hub genes between ALL central nervous system metastasis- and neuronal development-related DEGs. Subsequently, we performed GO term and KEGG pathway enrichment analyses for DEGs. Meanwhile, we predicted non-coding RNAs that may regulate the co-expression of DEGs. Finally, we identified hematologic and neurological diseases associated with co-expressed genes in the CTD.

The hub gene that related to neuronal development was found among ALL central nervous system metastasis-related DEGs in this study through the PPI network constructed. As we know, a variety of protein tyrosine kinases (PTKs) are present in the central nervous system of mammals, many of which play a role in the formation of neurons and the alteration of synapses (21). LCK is one of the five members of the Src family of non-receptor protein tyrosine kinases (22). Martinez’s group discovered that LCK can phosphorylate δ-catenin and bind to δ-catenin through its polyproline tract to regulate neurite growth (23). The *N*‐methyl‐d‐aspartate receptor (NMDAR), a ligand‐gated ion channel, plays a crucial role in controlling synaptic plasticity, brain development, and excitotoxicity within the central nervous system (24). Le’s study indicated that LCK may serve as an upstream to have impacts on NMDAR tyrosine phosphorylation, which mediated processes such as learning and memory and hippocampal neuron migration (25). Meanwhile, as a non-receptor tyrosine protein kinase, LCK is crucial for the selection and development of developing T cells, as well as the functioning of mature T cells. It has been documented that it is associated with the onset of acute lymphoblastic leukemia and appears to facilitate the metastatic characteristics of leukemic cells (26, 27). These findings imply that LCK may act as a bio-functional mediator to correlate neuronal development and ALL central nervous system metastasis.

Furthermore, we observed the presence of hub genes that are directly or indirectly involved in the regulation of hematologic malignant diseases within the differentially expressed genes associated with neuronal development. Laminins play a crucial role in a wide range of physiological and pathological processes, such as contributing to the formation of basement membranes and the growth of neurites and facilitating cell adhesion, migration, proliferation, and angiogenesis in cancer cells (28). The connection between cancer cells and laminins is essential for tumor spread and metastasis. The interaction between invading tumor cells and laminins leads to an increased potential for metastasis (29). Research has indicated that LAMB1 may be a viable biomarker for certain types of cancer (30, 31). Pan-cancer analysis showed the correlation between abnormal expression of LAMB1 in various cancers and clinical outcomes (32). LAMB1 plays a role in the movement and infiltration of cells into the adjacent extracellular matrix in prostate cancer (30). Moreover, hepatocellular carcinoma (HCC) exhibited an upregulation of LAMB1, which was associated with heightened tumor aggressiveness and unfavorable patient survival (33). Simultaneously, in the nervous system, it is believed that laminins play a role in several processes, including neurite outgrowth, neural survival, and synapse formation and function (34). Recent studies indicated that IGFBP3 could potentially inhibit tumor growth through induction of the tumor suppressor gene p53 (35). The subsequent occurrence of relapse in childhood leukemia was found to be inversely correlated with the rising levels of total IGFBP3 in a molecular mechanistic study (36). Likewise, multiple lines of evidence indicate that insulin-like growth factor-1 (IGF-1) is essential to normal brain growth and development (37). The regulatory action of IGF-1 is contingent upon the presence of IGFBP3, which has the ability to either impede or promote cellular proliferation. This outcome is determined by the expression of proteases that facilitate the release of IGF-1 from the IGF-1–IGFBP3 complex (38). Kalluri has demonstrated that IGFBP3 could regulate neural progenitor cell proliferation and reduce the amount of nestin in the neural progenitor cells, indicating its potential role in neuronal development (39). Galectins have been extensively linked to cancer, specifically controlling cell transformation, apoptosis, proliferation, migration, invasion, and angiogenesis (40), and LGALS1 has been previously documented to enhance the survival of hematological malignancies by directly targeting tumor cells (41). LGALS1 could promote the proliferation of adult neural stem cells (NSCs) found in several studies (42, 43). The role of hub genes LCK, LAMB1, IGFBP3, and LGALS1 in the pathogenesis and metastasis of acute lymphoblastic leukemia as well as in the development of the nervous system provides evidence to support our hypothesis that there may be a relationship between ALL central nervous system metastasis and neuronal development, which may be arise from locus mutations or gene variants.

The co-DEGs LGALS1, TMEM71, SHISA2, and S100A11 were identified in our study. LGALS1, a lectin that binds beta-galactoside and a wide array of complex carbohydrates, has a critical role in cell proliferation, cell differentiation, and ALL occurrence (41). TMEM71 is a member of an extensive gene family that encodes transmembrane (TMEM) proteins. In recent years, scholars have shown growing interest in the involvement of TMEMs in malignancies. For example, Wang et al. observed a significant correlation between elevated TMEM71 expression and reduced survival time among patients diagnosed with both glioma and glioblastoma (44). TMEM14A plays a role in the development and spread of ovarian cancer, TMEM88 has been documented to induce invasion and metastasis in breast cancer through its interaction with disheveled (Dvl) proteins, TMEM45A and TMEM71 facilitate the proliferation and invasion of glioma cells, and TMEM45B enhances the proliferation, migration, and invasion of gastric cancer cells through the JAK2/STAT3 signaling pathway (45). Nagano’s research demonstrated that SHISA2 is indispensable for the maturation of presomitic mesoderm cells (46). In a similar vein, there have been recent reports of its overexpression in high-grade prostate cancer cells and its involvement in the aggressive phenotype of prostate cancer (47). Research has demonstrated that S100A11 could be involved in movement, infiltration, and tubulin polymerization (48). Tumor metastasis has been linked to chromosomal rearrangements and modified gene expression (49). These co-DEGs play a role in both tumor metastasis and nervous system development, which further suggests that the occurrence of nervous system metastasis in acute lymphoblastic leukemia may be related to neuronal development.

MicroRNAs, a key component of the non-coding RNA family, are involved in multiple cellular functions. The development of miRNA delivery strategies for the treatment of cancer patients such as acute lymphoblastic leukemia was prompted by the emergence of the miRNA field. Cancer cells secrete miRNAs, and their abnormal expression is linked to tumor growth and advancement; these miRNAs are not only potential targets for treatment but also potential biomarkers for diagnosis and prognosis (50). We found that hsa-miR-22-3p, hsa-miR-548t-5p, and hsa-miR-6134 may be potential biomarkers of neuronal development-related ALL central nervous system metastasis. It is noteworthy that Saccomani’s team discovered that an increase in miR-22-3p expression in T-ALL cells can impede colony formation in a laboratory setting and the advancement of leukemia in a living organism (51). Li’s research showed that miR-22-3p had the ability to raise p-EGFR levels and lengthen neurites through the STAT3/GAP43 pathway (52). Additionally, miR-548t-5p and other non-coding RNAs could regulate cell proliferation and metastasis in multiple human cancers (53–55). As shown in **Table 2**, we revealed that miRNAs involved in hsa-miR-22-3p, hsa-miR-548t-5p, and hsa-miR-6134 are related to ALL central nervous system metastasis and neuronal development. These miRNAs may be considered potential biomarkers to develop novel diagnostic and therapeutic strategies in ALL central nervous system metastasis.

The CTD (https://ctdbase.org/) proposes to combine diverse data on chemical exposures and their biological effects. In the CTD, both the hematological diseases and nervous system diseases associated with the four co-DEGs are identified, which helps us develop hypotheses about mechanisms underlying ALL central nervous system metastasis related to neuronal development. In a translational view, we have applied a non-specific VEGF inhibitor (thalidomide) to evaluate whether interfering with the neuronal development process could hamper ALL cells from metastasizing to the CNS. The results showed a significant decrease in several neuronal development genes, which suggests a promising and novel therapy against CNS metastasis of ALL.

The present study provides several candidates for further research on mechanisms of ALL central nervous system metastasis pathogenesis. Notably, our results suggest that CNS infiltration in ALL may be caused by leukemia cells mimicking the process of neuronal development. Additionally, the co-DEG expression profile could be used to help diagnose, differentiate, predict, and design treatments for CNS metastasis in ALL. Additional research is necessary to validate our discoveries and clarify the mechanisms by which these potential biomarkers operate in the metastasis of the entire central nervous system.

The interpretation of our findings should be addressed with caution due to certain limitations. First, being largely limited to *in silico* evaluation, our findings should be verified and extended in laboratory experiments. Second, given that the microarray analysis was conducted using gene expression rather than protein expression, the biomarkers used in this study should be regarded as genes rather than proteins. We may be able to validate our results to some extent by conducting larger and more prospective clinical studies.

## Conclusion

Hub genes CD2, CD3G, CD3D, and LCK may be associated with ALL central nervous system metastasis; LAMB1, MATN3, IGFBP3, LGALS1, and NEUROD1 may be associated with neuronal development. Additionally, co-DEGs of LGALS1, TMEM71, SHISA2, and S100A11 link ALL central nervous system metastasis and neuronal development. Additionally, the miRNAs for each co-DEG may be potential biomarkers or therapeutic targets for ALL central nervous system metastasis, especially hsa-miR-22-3p, hsa-miR-548t-5p, and hsa-miR-6134. Thus, we hypothesized that there may be an association between ALL central nervous system metastasis and neuronal development and that expression of LGALS1, TMEM71, SHISA2, and S100A11 genes that favor ALL central nervous system metastasis is related to neuronal development.

## Data availability statement

The original contributions presented in the study are included in the article/**Supplementary Material**. Further inquiries can be directed to the corresponding authors.

## Ethics statement

The studies involving humans were approved by Ethics Committee of Tongji Medical College, Huazhong University of Science and Technology. The studies were conducted in accordance with the local legislation and institutional requirements. The participants provided their written informed consent to participate in this study. Written informed consent was obtained from the individual(s) for the publication of any potentially identifiable images or data included in this article.

## Author contributions

ZL and ZG: data analysis and writing the manuscript. HX and XC: experiments execution and data analysis. HZ and WL: designing, project administration, supervision. All authors reviewed the manuscript.

## Glossary

**Table:**

ALL	acute lymphoblastic leukemia
CNS	central nervous system
DEGs	differentially expressed genes
co-DEGs	co-expressed differentially expressed genes
miRNAs	microRNAs
GEO	Gene Expression Omnibus
PPI	protein–protein interaction
GO	Gene Ontology
OS	overall survival
BBB	blood–brain barrier
BTB	blood–tumor barrier
CSF	cerebrospinal fluid
NPCs	neural progenitor cells
KEGG	Kyoto Encyclopedia of Genes and Genomes
DAVID	Database for Annotation, Visualization and Integrated Discovery
BP	biological process
MF	molecular function
CC	cellular component
mirDIP	microRNA Data Integration Portal
miRDB	microRNA Target Prediction Database
CTD	The Comparative Toxicogenomics Database
LGALS1	galectin 1
TMEM71	transmembrane protein 71
SHISA2	shisa family member 2
S100A11	S100 calcium-binding protein A11
LCK	lymphocyte-specific protein tyrosine kinase
IL2RB	interleukin 2 receptor subunit beta
LAMB1	laminin subunit beta 1
MATN3	matrilin 3
IGFBP3	insulin-like growth factor binding protein 3
NEUROD1	neuronal differentiation 1
MAP	mitogen-activated protein
PTKs	protein tyrosine kinases
NMDAR	*N*-methyl-d-aspartate receptor
HCC	hepatocellular carcinoma
IGF-1	insulin-like growth factor-1
NSCs	neural stem cells
TMEM	transmembrane
Dvl	disheveled
