# Supplementary material for: Simulating neuronal development: exploring potential mechanisms for central nervous system metastasis in acute lymphoblastic leukemia
Source: Front Oncol. 2024 Jan 4;13:1331802. doi: 10.3389/fonc.2023.1331802 (PMC10794646; doi:10.3389/fonc.2023.1331802)
Supplement: Supplementary file 1 [file DataSheet_1.pdf]

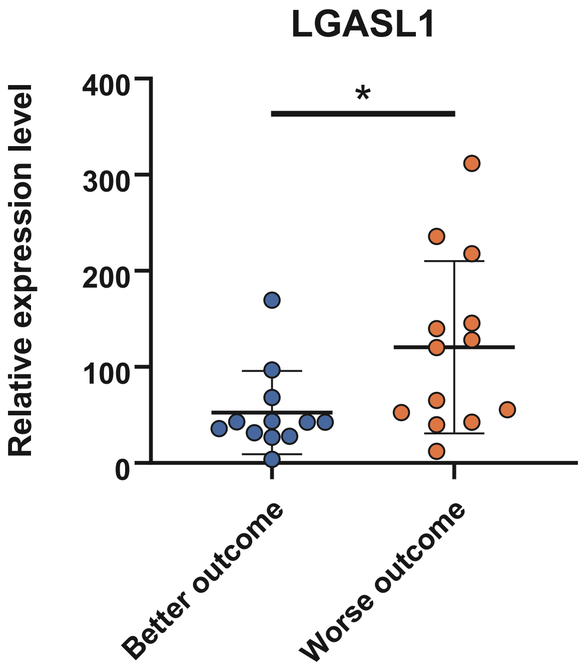

Supplementary Figure 1: GEO data analysis (GSE5820) revealing LGALS1 is upregulated in ALL patients with worse clinical outcome.
