# Supplementary material for: Simulating neuronal development: exploring potential mechanisms for central nervous system metastasis in acute lymphoblastic leukemia
Source: Front Oncol. 2024 Jan 4;13:1331802. doi: 10.3389/fonc.2023.1331802 (PMC10794646; doi:10.3389/fonc.2023.1331802)
Supplement: Supplementary file 7 [file Table_6.docx]

| **Characteristics** | **Patients (N=10)** |
| --- | --- |
| **Age (y)** | 18-57 |
| **Gender (M/F)** |  |
| Male (M) | 7 |
| Female (F) | 3 |
| **Cytogenetic abnormalities** |  |
| BCR/ABL Positive | 4 |
| BCR/ABL Negative | 6 |
| **Fever/infection** | 6 |
| **Pallor** | 5 |
| **Bleeding tendency** | 2 |
| **Splenomegaly** | 7 |
| **Lymphadenopathy** | 9 |
| **White blood cell count, ×10^9^/L** | 8.1-19.3 |
| **Hemoglobin, g/L** | 63-101 |
| **Platelet count, ×10^9^/L** | 13.5-148.7 |
| **Neutrophil count, ×10^9^/L** | 1.2-3.8 |
| **Lymphocyte count, ×10^9^/L** | 6.4-17.2 |
| **Albumin, g/L** | 30-46 |
| **Creatinine, µmmol/L** | 71-132 |

**Table S6.** **Baseline characteristics of ALL patients with CNS infiltration.**
